# The effects of equine peripheral blood stem cells on cutaneous wound healing: a clinical evaluation in four horses

**DOI:** 10.1111/ced.12068

**Published:** 2013-03-21

**Authors:** J H Spaas, S Broeckx, G R Walle, M Polettini

**Affiliations:** 1 Department of Comparative Physiology and Biometrics, Faculty of Veterinary Medicine, Ghent UniversityMerelbeke, Belgium; 2 Department of Reproduction, Obstetrics and Herd Health, Faculty of Veterinary Medicine, Ghent UniversityMerelbeke, Belgium; 3 Private Practitioner in Veterinary MedicineSutri Viterbo, Rome, Italy

## Abstract

Stem-cell therapy represents a promising strategy for the treatment of challenging pathologies, such as large, infected wounds that are unresponsive to conventional therapies. The present study describes the clinical application of peripheral blood stem cells (PBSCs) for the treatment of four adult Warmblood horses with naturally occurring wounds, which were unresponsive to conventional therapies for at least 3 months. A visual assessment was performed, and a number of wound-healing parameters (granulation tissue, crust formation and scar formation) were evaluated. In all cases, tissue overgrowth was visible within 4 weeks after PBSC injection, followed by the formation of crusts and small scars in the centre of the wound, with hair regeneration at the edges. In conclusion, this is the first report of PBSC therapy of skin wounds in horses, and it produced a positive visual and clinical outcome.

Extended wounds of the distal limbs are one of the most common dermatological pathologies in horses, and tend to have a long recovery period and poor response to conventional therapies.^1^–^4^ In horses, secondary-intention wound healing occurs significantly more slowly in metatarsal wounds than in other wounds such as muscle wounds.^1^–^4^ This difference reflects the contribution of contraction to wound healing. Demarcation is also seen later and a healthy granulation bed develops more slowly, because wounds remain irregular and more purulent for a longer time in horses than in most animal species.^1^–^4^ Another reason for the extended healing period is the slow epithelialization process.^4^,^5^ Because horses have a weak initial inflammatory response, the natural wound debridement process is slow, and there is a high risk of infection.^6^–^8^ However, after the initial phase, horses have the tendency to develop chronic inflammation at the site of the wound, which causes excessive granulation tissue.^6^,^8^,^9^ Therefore, wound infection or exuberant granulation are the origin of the delayed healing time in most cases. Currently, skin grafting, using grafts from other parts of the body, is the treatment of choice,^10^ although accessory skin loss, infection, and difficult attachment to the underlying and surrounding tissues are major complications, reducing the chance of success of skin transplantation in this animal species.^1^,^2^,^11^,^12^ Because allogeneic skin graft rejection is a well-known risk in humans,^13^ skin tissue taken from other horses has, to our knowledge, been used to date only as a biological dressing.^14^ Hence, because of the aforementioned difficulties in wound healing in horses, the use of regenerative therapies has recently been suggested as a promising new treatment for equine dermatological pathologies.^15^

It is known that during the inflammatory phase of the wound-healing process, macrophages release biologically active substances (cytokines), which are essential for the recruitment of various types of blood cells, such as inflammatory cells, mesenchymal cells^16^ and peripheral blood stem cells (PBSCs).^17^ All these cells take part in the formation of granulation tissue by producing extracellular-matrix components and by simulating wound contraction. Therefore, we hypothesized that injecting PBSCs directly into the wound might be of use for the treatment of skin wounds that are unresponsive to conventional therapies. Interestingly, bone marrow-derived mesenchymal stem cells (MSCs) have been described to increase the healing capacity of persistent leg wounds in human patients with diabetes.^18^ However, no dermatological pathologies in horses have been treated with stem cells to date, although peripheral blood has recently been described as an attractive source of stem cells in horses (minimally invasive, non-painful and easily harvested).^19^,^20^ In the present study, we evaluated the clinical effects of equine PBSCs on different types of naturally occurring wounds on the limbs of four horses, which were unresponsive to conventional therapies.

## Methods

The study was approved by the local ethics committee (approval EC2010-147), and all procedures were carried out with due care for animal welfare.

### Horses

Four horses were enrolled in the study. All had sustained injuries to the legs.

**Figure 1:**
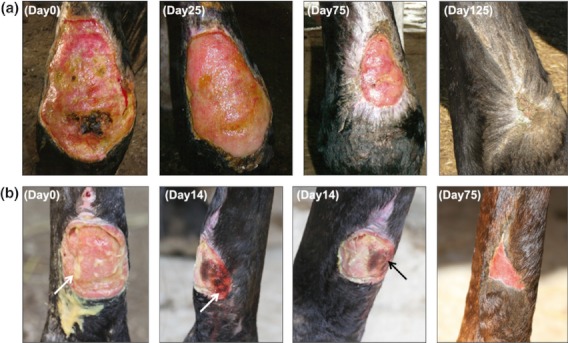
Representative photos of the wounds of (a) an 11-year-old mare (a) and (b) a 16-year-old gelding on the day of therapy (day 0) with peripheral blood stem cells (PBSCs) and at three different time points after PBSC therapy, showing granulation tissue, crust formation and scar formation, respectively.

An 11-year-old mare sustained a skin wound to the dorsal surface of the metatarsal bone, which subsequently became infected with *Clostridium* species (Fig. 1a). After wound debridement, removal of the digital extensor tendon, and conventional medical treatment consisting of broad-spectrum antibiotics in combination with non-steroidal anti-inflammatory drugs, there was no improvement in the wound. Therefore, the same surgical and medical treatment were repeated; however, the extended wound persisted for 3 months, and at one point the underlying bone was visible through the necrotic tissue.

A 16-year-old gelding had a skin wound at the plantar surface of the metatarsal bone (Fig. 1b), which did not heal after 3 months of conventional therapy, including wound debridement, local wound-healing creams and general antibacterial therapy. Moreover, the wound surface was covered with pus, which is commonly associated with a bacterial infection.

**Figure 2:**
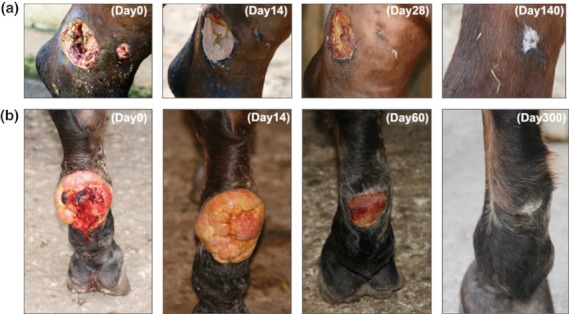
Representative photos of the wounds of two 26-year-old geldings, case reports (a) 3 and (b) 4, on the day of therapy (day 0) with peripheral blood stem cells (PBSCs) and at three different time points after PBSC therapy, showing granulation tissue, crust formation and scar formation, respectively.

A 26-year-old gelding sustained a deep wound with bone exposition at the medial surface of the tibia (Fig. 2a). Because the wound was not responsive to wound debridement and medical treatment for 3 months, stem-cell therapy was recommended as a last resort.

Another 26-year-old gelding had a wound on the leg for 3 months. Non-neoplastic exuberant granulation was seen on the plantar surface of the metatarsal bone (Fig. 2b). Nodular proliferative lesions were present, which tended to recur after surgery (resection of the granulation tissue and wound debridement) and to form ulcers. Treatment with broad-spectrum antibiotics for 30 days did not improve the symptoms.

### Preparation of peripheral blood stem cells

Samples (5–7 mL) of autologous blood were collected from the jugular vein of the injured horses into sterile tubes containing EDTA. The nucleated blood-cell fraction was separated by incubation with ammonium chloride (dilution 1 : 3 in 1 mol/L ammonium chloride), followed by centrifugation at 400 ***g***. The separated fraction was washed several times with phosphate-buffered saline (Sigma–Aldrich, St Louis, MO, USA) to remove most of the erythrocyte fraction, as previously described.^16^ Cells were then incubated for 72 h at 37 °C in the presence of 50 nmol/L macrophage colony-stimulating factor and 5 μmol/L gentamicin (both Sigma–Aldrich). Subsequently, the cells were incubated for 30 min at room temperature with saturating amounts of fluorescently labelled human antibodies (either alone or in combination) against various markers that crossreact with horse antigens. Cells were then sorted by fluorescence-activated cell sorting (FACSAria II; BD Biosciences Inc., San Jose, CA, USA) confirming the presence of PBSCs, which were positive for peridinin chlorophyll-conjugated CD34 and allophycocyanin-conjugated CD117 (haematopoietic stem-cell markers) and for phycoerythrin-conjugated CD90 and fluorescein isothiocyanate-conjugated CD105 (mesenchymal stem-cell markers) (all from BD Biosciences).

### Treatment and assessment of healing

After approximately 3 days, 5 × 10^5^ and 1.25 × 10^5^ PBSCs were each resuspended in 2 mL of PBS for local and intravenous injection, respectively. The autologous PBSCs, kept at 4–7 °C, were transported to the animals within 24 h. Local (intradermal) injections were made into 5–6 different locations at the wound edges, and an intravenous injection was made into the jugular vein. Afterwards, the peripheral wound edges were surgically prepared with an iodine solution and alcohol. Detomidine (0.04 mg/kg; Domosedan®; Janssen Animal Health, High Wycombe, Buckinghamshire, UK) and butorphanol (0.1 mg/kg; Torbugesic®; Fort Dodge Animal Health, Fort Dodge, IA, USA) were intravenously administered (using a 21 G needle measuring 40 mm) as sedative and analgesia.

The *in vivo* efficacy was assessed by evaluating hypersensitivity reactions and weal formation (physical reaction) after injection with PBSCs. Various wound-healing parameters (granulation tissue, crust formation and scar formation) were visually evaluated daily, and the time points of their appearance were documented until no more progress could be noticed for at least 3 weeks (Table 1). A monthly follow-up was performed until 1 year after injection.

**Table 1:** Time points of appearance of different wound healing parameters in days after treatment with autologous peripheral blood stem cells (PBSCs)

	Days after treatment with PBSCs		

Animal no.	Granulation tissue	Crust formation	Scar formation
1	25	75	125
2	14	14	75
3	14	28	140
4	14	60	300
Mean ± SD	17 ± 6	44 ± 28	160 ± 97

### Statistical analysis

Data were analysed using the Student *t*-test for group comparisons of normally distributed variables. Values are given as means ± SD. *P*-values were calculated using an Excel spreadsheet (2007; Microsoft Corp., Redmond, WA, USA), and *P* < 0.05 was considered significant.

## Discussion

We report the use of PBSCs to treat long-lasting skin wounds in horses, which were unresponsive to conventional medical and surgical treatments. In cases of ongoing bacterial infection, antibiotic treatment was continued. No hypersensitivity reactions or weal formation (physical reaction) were seen in any of the four animals after injection of PBSCs, which is in contrast to a previous report describing weal formation after injection of umbilical cord-derived MSCs in Quarterhorse yearlings.^21^

Because epithelialization is a slow process in horses, it usually takes months before a large wound is fully healed.^22^ In particular, metatarsal wounds in horses differ markedly from that of all other wounds: these wounds increase to almost twice their original size in the first 2 weeks, exuberant granulation tissue is persistent, and epithelialization starts later than in wounds on other parts of the body.^3^ In the present study, granulation tissue began forming within 4 weeks of the PBSC therapy in all four cases, which was a significant (*P* < 0.001; Student *t*-test) improvement compared with the previously failed conventional therapies, which had been used for at least 3 months (Table 1). Furthermore, crust formation was achieved within 2 months, which was also significantly (*P* < 0.05) faster than had been achieved with the failing conventional therapies (Table 1). Because it took longer than 3 months after PBSC therapy for scars to form in three of the four cases, this wound-healing parameter showed no significant difference between conventional and PBSC therapy (Table 1). However, none of the animals had any residual swelling or lameness after full recovery, and eventually only small scars were formed in all four cases (Figs 1 and 2). This is in contrast to a previous study,^23^ in which similar wounds resulted in permanently swollen legs in combination with large scar formation and long-lasting lameness after treatment with various conventional therapies. In the present study, none of the animals had swollen leg after PBSC therapy, and there were no signs of lameness once crust formation had taken place. In addition, we found that for animal four, the granulation tissue could be easily removed without recurrence of the wound (Fig. 2b), which is also in contrast to the results with the previous conventional treatment this horse received. At follow-up 1 year after PBSC therapy, none of the cases showed wound recurrence or other adverse effects.

In conclusion, we report the use of PBSC therapy for large wounds on the legs of four horses. Despite previous failure of conventional therapies, all four animals responded well to the PBSC therapy, indicating that this might be a useful treatment for large wounds in horses. Further studies using a larger numbers of animals, a double-blind standardized model and appropriate controls are warranted to confirm the results of this small-scale study.

**Learning points**

- PBSC s can be locally applied by using multiple intradermal injections into the edges of naturally occurring chronic wounds in horses.
- An intravenous injection of PBSCs can also be performed in horses without any noticeable adverse effects.
- When wounds are infected, PBSC therapy is still an option, in combination with antibacterial medication.
- We found that in one animal, granulation tissue could be easily removed after PBSC therapy without any recurrence, which was in contrast to the previous conventional treatment this horse received, when removal of the granulation tissue led to a chronic recurrence of the exuberant granulation.
- At follow-up 1 year after PBSC therapy, there was no wound recurrence or other adverse effects.
- Based on the lack of any clinical improvement with conventional treatment for 3 months in these horses, the results of this case study show the promising application of PBSCs for accelerating the healing of damaged skin.
